# Risk factors for choroidal detachment following rhegmatogenous retinal detachment in a chinese population

**DOI:** 10.1186/s12886-016-0319-9

**Published:** 2016-08-09

**Authors:** Yajie Yu, Ming An, Bin Mo, Zhen Yang, Wu Liu

**Affiliations:** 1 Beijing Tongren Eye Center, Beijing Tongren Hospital, Capital Medical University, Beijing Ophthalmology & Visual Sciences Key Laboratory, Beijing, 100730 China; 2 Department of Ophthalmology, Qingdao Municipal Hospital, Qingdao, Shandong 266011 China; 3 Department of Ophthalmology, the second people’s hospital of Ji’nan city, Ji’nan, 250022 China

**Keywords:** Risk factor, Retinal detachment, Choroidal detachment, Macular hole

## Abstract

**Backgroud:**

Choroidal detachment (CD) following primary rhegmatogenous retinal detachment (RRD) is a special type of RRD. The purpose of this study is to investigate the potential risk factors of RRD with CD in a Chinese population.

**Methods:**

All of 201 consecutive RRD with CD patients and 210 RRD without CD patients were enrolled in this case–control retrospective study. The clinical data from these cases were reviewed here. Patients were undergone scleral buckling or encircling or both, or pars plana vitrectomy with or without scleral buckling or encircling or both according the patients’ condition. The incidence of RRD with CD in this Chinese population was measured, and the potential risk factors for the development of RRD with CD were investigated by multivariate logistic regression analysis.

**Results:**

In this population, the incidence of RRD with CD was 8.6 %. The incidence of RRD with CD was significantly higher in patients with macular hole (*P <* 0.05), retinal breaks located posterior to the equator (*P <* 0.05), and total detachment (*P <* 0.05). Furthermore, the incidence of RRD with CD was significantly higher in patients with longer axial length (*P <* 0.05) only when ages, IOP, AL and duration time was set for categorical variables.

**Conclusions:**

Hypotony, retinal breaks located posteriorly especially macular hole, longer axial length, and the whole retinal detachment might be the potential risk factors for the development of CD in RRD patients.

## Background

The worldwide occurrence of choroidal detachment (CD) following primary rhegmatogenous retinal detachment (RRD) is rare, affecting only 2 %–4.5 % of RRD cases [1–3], but the incidence is higher (1.5 %–18.1 %) in China [4]. This special form of RRD, first reported in 1974 [2], is associated with choroidal and ciliary body detachment and characterized by rapid progression, poor visual prognosis, and poor treatment options [5]. Because of the high incidence of proliferative vitreoretinopathy after surgery,, varying from 35.4 %–52.4 %, [3, 6] the results of conventional scleral buckling in eyes with RRD and CD have been reported to be relatively poor. This unfavorable prognosis has been attributed to poor visualization, difficult identification of causative breaks, difficult application of retinopexy, and the development of proliferative vitreoretinopathy. Although the treatment success of RRD with CD has been improved with the introduction of a vitrectomy technique [7–9] and perioperative pharmacological management [9–11], CD is still considered to be a preoperative risk factor for the failure of RRD repair.

In most cases, CD occurs after the development of RRD. In previous studies, CD was reported to occur more frequently in RRD patients with high myopia, aphakia, pseudophakia, and advanced age, who usually present with low intraocular pressure (IOP) and multiple retinal breaks [2, 12]. A correlation of macular hole [13] with the development of CD in RRD patients was also found. However, due to the small sample size, these studies were inconclusive.

The purpose of this case–control retrospective study was therefore to identify, using a large sample size, the potential risk factors for the development of CD in RRD.

## Methods

Patients that underwent scleral buckling or pars plana vitrectomy (PPV) for retinal reattachment at the Department of Ophthalmology of Beijing Tongren Hospital affiliated with Capital Medical University from September 2009 to August 2014 were identified from medical records. Cases that underwent initial surgery for RRD treatment at this hospital were included. Exclusion criteria included recurrent retinal detachment after surgery, diabetes, hypertension and other systemic diseases, proliferative diabetic retinopathy, wet age-related macular degeneration, ischemic central/branch retinal vein occlusion and other eye diseases that required intravitreous injection of anti-VEGF drugs, and RRD development resulting from penetrating ocular trauma or intraocular surgery.

Due to RRD with CD only contributing a minor part of RRD, patients in RRD without CD with matched gender and age with patients in RRD with CD were randomly selected by table of random number. And then, whether gender and age matched between two groups was respectively verified by chi-square test and paired *t* test before multivariate logistic regression analyses.

This study adhered to the tenets of the Declaration of Helsinki and the ARVO Statement on Human Subjects. It was approved by the Ethics Committee of Beijing Tongren Hospital affiliated with Capital Medical University. Informed consent using a form approved by the Institutional Review Board was obtained from each patient before surgery.

All cases were identified using the databases in the Medical Records Reading Room at Beijing Tongren Eye Center. The clinical data from these cases were reviewed to confirm case status and ascertain risk factor information.

Patients’ demographics, including gender, age, and RRD duration were recorded. All patients received detailed ophthalmic examinations, including best-corrected visual acuity (BCVA), IOP, anterior segment evaluation with a slit-lamp microscope, funduscope examination with a binocular indirect ophthalmoscope, and B ultrasound performed by experienced ophthalmologists. Ultrasonic biomicroscopy examination was also used to provide objective visual evidence for RRD with CD, and the type, number, and location of the retinal breaks found during surgery were recorded in detail.

Statistical analyses were performed using SPSS, version 17.0 (SPSS, Chicago, IL, USA). Multivariate logistic regression analyses were used to investigate the potential risk factors for CD following RRD.

## Results

A total of 2,348 consecutive cases that underwent scleral buckling or PPV for retinal reattachment at the Department of Ophthalmology at Beijing Tongren Hospital affiliated with Capital Medical University, (including 201 RRD with CD and 2,147 RRD without CD patients) were reviewed in this study. The incidence of RRD with CD in this population was 8.6 % (201/2,348). All 201 patients with RRD with CD patients, and 210 patients with RD and no CD, who were randomly selected from 2,147 consecutive patients, were enrolled in this study. And no significant differences in both gender and age were found between two groups (*P >* 0.05).

Patient demographics and clinical parameters are shown in Table 1. A high incidence of low IOP (IOP < 7 mmHg) and total retinal detachment in the RRD with CD group was noted, with no obvious trends for the other variables.

**Table 1:** The characteristic information for RRD with CD patients and RRD without CD patients

Charactristic	RRD with CD (*n =* 201)	RRD without CD (*n =* 210)
Sex		
Male	107 (53.23 %)	109 (51.90 %)
Female	94 (46.77 %)	101 (48.10 %)
Age (y)		
≥50	96 (47.76 %)	105 (50.00 %)
<50	105 (52.24 %)	105 (50.00 %)
Visual acuity		
≥4/200	52 (25.87 %)	94 (44.76 %)
<4/200	149 (74.13 %)	116 (55.24 %)
IOP (mmHg)		
≥7	127 (63.18 %)	202 (96.19 %)
<7	74 (36.82 %)	8 (3.81 %)
Axial length (mm)		
≥24 mm	120 (59.70 %)	135 (64.29 %)
<24 mm	81 (40.30 %)	75 (35.71 %)
Lens		
Phakic	179 (89.05 %)	194 (92.38 %)
Aphakic/pseudopakic	22 (10.95 %)	16 (7.62 %)
Duration time (d)		
≥40	127 (63.18 %)	139 (66.19 %)
<40	74 (36.82 %)	71 (33.81 %)
Retinal detachment (extent)		
≤3 quadrants	11 (5.47 %)	154 (73.33 %)
4 quadrants	190 (94.53 %)	56 (26.67 %)
Retinal break		
Number		
1	113 (56.22 %)	132 (62.86 %)
≥2	88 (43.78 %)	78 (37.14 %)
Type		
Macular hole	34 (16.92 %)	16 (7.62 %)
Atrophic	51 (25.37 %)	98 (46.67 %)
Traction	104 (51.74 %)	74 (35.24 %)
Compound	12 (5.97 %)	22 (10.47 %)
Location		
EQ/AEQ	146 (72.64 %)	184 (87.62 %)
PEQ	42 (20.89 %)	21 (10.00 %)
Both	13 (6.47 %)	5 (2.38 %)

IOP = intraocular pressure; EQ = equator; AEQ = anterior to equator; PEQ = posterior to equator

The results of multivariate logistic regression analyses are shown in Table 2. When the age, IOP, axial length (AL), and RRD duration were set as continuous variables, significant differences were found in IOP, extent of retinal detachment, and the type and location of the retinal breaks. Furthermore, the incidence of RRD with CD in RRD patients decreased by 20 % with every one mmHg increase in IOP (*P =* 0.001). When compared with traction holes, the incidence of CD among RRD patients was significantly higher among those with macular holes (*P =* 0.000), atrophic holes (*P =* 0.027), and compound holes (*P =* 0.005). When compared with retinal breaks located anterior to or just on the equator, the incidence of RRD with CD in RRD patients was also significantly higher in retinal breaks located posterior to the equator. When compared with whole retinal detachment, the incidence of RRD with CD in RRD patients was significantly lower in patients whose extent of retinal detachment was ≤ 3 quadrants (*P =* 0.000).

**Table 2:** The results of the multivariate logistic regression analysis when ages, IOP, AL and duration time were set for continuous variables

	B	S.E.	Wald	Odds Ratio (95 % Confidence Interval)	*P* Value
Sex (female)	0.21	0.52	0.17	1.24 (0.45–3.39)	0.682
Age	–0.01	0.02	0.11	0.99 (0.96–1.03)	0.736
Duration time	–0.01	0.00	2.29	1.00 (0.99–1.00)	0.130
Visual acuity(≥4/200)	–0.01	0.59	0.00	0.99 (0.31–3.13)	0.983
IOP	–0.23	0.07	10.23	0.80 (0.70–0.92)	0.001
Axial length	0.09	0.09	0.83	1.09 (0.91–1.31)	0.362
Lens (Aphakic/pseudopakic)	0.12	0.75	0.02	1.122 (0.26–4.89)	0.878
Retinal break					
Number (≥2)	0.24	0.70	0.12	1.27 (0.32–5.00)	0.732
Type					
Traction hole				1 (reference)	
Macular hole	5.45	1.46	13.96	233.65 (13.37–4084.28)	0.000
Atrophic hole	2.72	1.23	4.91	15.15 (1.37–167.80)	0.027
Compound hole	3.71	1.32	7.91	41.03 (3.08–546.20)	0.005
Location					
EQ/AEQ				1 (reference)	
PEQ	6.16	0.93	43.52	472.38 (75.81–2943.27)	0.000
Both	24.00	9258.60	0.00	26521638949.823	0.998
Extent (total detachment)	–3.49	0.71	24.23	0.03 (0.01–0.12)	0.000

*IOP* intraocular pressure, *EQ* equator, *AEQ* anterior to equator, *PEQ* posterior to equator

Multivariate logistic regression analyses with age, IOP, AL, and duration as categorical variables are shown in Table 3. Significant differences were found not only in IOP, extent of retinal detachment, and the type and location of retinal breaks, but also for AL. When compared with a high IOP (IOP ≥ 7 mmHg), the incidence of CD among RRD patients was significantly higher (*P =* 0.008) in patients with low IOP (IOP < 7 mmHg). When compared with patients with longer AL (AL ≥ 24 mm), the incidence of CD among RRD patients was significantly lower (*P =* 0.011) in patients with lower AL (AL < 24 mm). When compared with patients with traction holes, the incidence of CD among RRD patients was significantly higher (*P =* 0.031) in patients with macular holes, but significantly lower in patients with compound holes (*P =* 0.002). Although there was no significant difference, the incidence of CD among RRD patients was slightly lower (*P =* 0.143) in patients with atrophic hole compared with patients with traction holes. When compared with patients with retinal breaks located anterior to or just on the equator, the incidence of CD among RRD patients was also significantly higher in retinal breaks located posterior to the equator (*P =* 0.000). When compared with patients with whole retinal detachments, the incidence of CD among RRD patients was also significantly lower in patients whose extent of retinal detachment was ≤ 3 quadrants (*P =* 0.000).

**Table 3:** The results of the multivariate logistic regression analysis when ages, IOP, AL and duration time were set for categorical variables

	B	S.E.	Wald	Odds Ratio (95 % Confidence Interval)	*P* Value
Sex (female)	0.50	0.54	0.83	1.64 (0.57–4.75)	0.361
Age (≥50y)	–0.15	0.54	0.08	0.86 (0.30–2.50)	0.783
Duration time (≥40d)	0.56	0.51	1.19	1.75 (0.64–4.78)	0.275
Visual acuity (≥4/200)	0.2	0.59	0.11	1.22 (0.38–3.90)	0.739
IOP (≥7 mmHg)	1.92	0.73	7.02	6.83 (1.65–28.28)	0.008
Axial length (≥24 mm)	–1.68	0.66	6.51	0.19 (0.05–0.68)	0.011
Lens (Aphakic/pseudopakic)	–0.09	0.86	0.01	0.91 (0.17–4.92)	0.914
Retinal break					
Number(≥2)	0.60	0.72	0.69	1.82 (0.44–7.48)	0.405
Type					
Traction hole				1 (reference)	
Macular hole	1.48	0.69	4.67	4.40 (1.15–16.90)	0.031
Atrophic hole	–1.03	0.71	2.15	0.36 (0.09–1.42)	0.143
Compound hole	–4.11	1.35	9.27	0.02 (0.00–0.23)	0.002
Location					
EQ/AEQ				1 (reference)	
PEQ	7.01	0.98	51.74	1109.22 (164.16–7494.87)	0.000
Both	25.14	8483.25	.000	82493234417.340	0.998
Extent (total detachment)	–3.56	0.74	23.418	0.03 (0.01–0.12)	0.000

*IOP* intraocular pressure, *EQ* equator, *AEQ* anterior to equator, *PEQ* posterior to equator

## Discussion

Although the pathogenic mechanism of CD following RRD has not yet been clearly delineated, most investigators believe that hypotony [14] induced by retinal detachment may induce choroidal detachment by arteriolar dilation and ciliary body edema and detachment.

Hypotony induced by retinal detachment is the first stage of choroidal detachment [14]. Liquefied vitreous enters the subretinal space through sensory retinal defects and stimulates choroidal abnormalities, including dilatation and hyperpermeability of choroidal vessels. The gradually increased exudation of fluid into the suprachoroidal and supraciliaris spaces results in choroidal detachment. In addition, edema of the ciliary body further reduces generation of aqueous humor and causes prominent hypotony [15], deteriorating the dilatation and hyperpermeability of choroidal vessels, and leading to progression of choroidal detachment. Therefore, a positive feedback loop is established.

In the present study, two different classification methods of multivariate logistic regression analysis both showed that hypotony was a risk factor for CD following RRD. When using IOP as a continuous variable, the incidence of CD in RRD patients decreased by 20 % with every one mmHg increase in IOP. When using IOP was converted to a categorical variable by separating it into two groups, ≥ 7 mmHg and < 7 mmHg, the incidence of CD among RRD patients was 6.83 times more in the latter group than in the former group.

Regardless of inflammation, the incidence of RRD with CD will increase, accompanied by a period of retinal detachment. With persistent hypotony, the dilation of choroidal vessels can result in leakage of the choroidal vessels into the suprachoroidal space, leading to choroidal detachment. Therefore, duration of retinal detachment might be an important risk factor for RRD with CD. However, in the present study, no significant difference in duration of detachment between RRD with CD versus RRD without CD was found. This lack of difference may result from the difference between the true (from the moment that retinal detachment occurs) and recorded (from the onset of symptoms) durations of RRD. Except in cases of severe retinal detachment, patients present no symptoms in the early stages, so the duration recorded in this study was much shorter than the real duration. In addition, Table 1 shows that the incidence of macular holes was much higher for RRD with CD than for RRD without CD (16.92 % versus 7.62 %, respectively). Due to the clinical characteristics of macular holes, the onset of detachment can be accurately described. Therefore, the duration in RRD with CD will be more accurate, while the reported duration of RRD without CD may be shorter than the real duration. This might lead to an insignificant difference in duration times for the two groups, and eliminate duration as a risk factor for RRD with CD.

Duration can also be affected by the transfer time of patients. The Department of Ophthalmology at Beijing Tongren Hospital affiliated with Capital Medical University is one of the best ophthalmology centers in China, so a large number of patients with retinal detachment transfer to this center from primary hospitals. The time for transferring both the RRD with CD and RRD without CD patients will therefore prolong the duration of detachment.

Previous reports of RRD with CD showed that risk factors for choroidal detachment include aphakia or pseudophakia, old age, and high myopia [2, 12]. However, in the present study, aphakia or pseudophakia and age were shown by multivariate logistic regression not to be risk factors. Here, all patients with RD were reviewed, including patients younger than 50 years of age, while in previous studies, most of the patients selected were older. This difference may result in differences in outcomes. For the same reason, because younger patients were included in the present study, the proportion of patients with aphakia or pseudophakia was lower than that in previous studies. Aphakia or pseudophakia was therefore not found to be a risk factor for RRD with CD.

Regarding myopia, because some patients underwent cataract extraction, the diopters of the aphakic or pseudopakic eyes were incorrect. In the present study, the AL, a parameter associated with myopia, especially high myopia, but not diopter, was used in our study. In high myopia, the diopter is more than -6 with axial myopia, which accompanies retinochoroidal atrophy in the majority of cases. In general, the AL increases about one mm when the degree of myopia increases by every -3 diopters. With increasing AL length in high myopia, retinal and choroidal tissues, including retinal pigment epithelium, will suffer from traction and trophic degeneration. Meanwhile, liquefied vitreous can enter into the subretinal space through the retinal breaks to form a large amount of subretinal fluid. Subretinal fluid absorption facilitated by the retinal pigment epithelial pump will result in severe hypotony in retinal detachment during high myopia. In addition, fundus degeneration decreases the ability of choroidal blood vessels to counter the changes of IOP. Hypotony induced by retinal detachment is more likely to cause increased flow of choroidal vessels, slower blood flow, flow stasis, and a further increase in choriodal detachment.

Consistent with a previous study [13], we also found that macular hole is a risk factor for RRD with CD. Based upon our findings, the location of retinal breaks and the posterior vitreous detachment might be the primary reasons for the high incidence of RRD with CD. In a representative progression of posterior vitreous detachment, the vitreous cortex separates from the retina first at the posterior pole, including the macular area, progressing in the anterior direction until it reaches the posterior margin of the vitreous base. In cases involving only peripheral retinal breaks, the breaks may be blocked by the covering vitreous gel, limiting the progression of retinal detachment, or even resulting in spontaneous retinal reattachment. In cases of RRD secondary to macular hole, usually accompanied by various degrees of posterior vitreous detachment, the covering vitreous gel above the macular hole is absent and the macular hole remains persistently open. Therefore, liquefied vitreous enters in an uncontrolled manner into the subretinal space through the macular hole, where it can be absorbed by the powerful retinal pigment epithelium pump. With the increasing extent of retinal detachment with macular hole, outflow through the retinal pigment epithelium may increase, resulting in a further decrease of intraocular pressure and choroidal detachment.

There is a contradictory place in the risk factor for the type of the retinal hole when the age, IOP, AL, and RRD duration were set as continuous variables or as categorical variables. As these variables were set as continuous variables, the incidence of CD among RRD patients was significantly higher among those with atrophic holes and compound holes while slightly lower among those with atrophic holes and significant lower among compound holes as these variables were set as categorical variables. This inconsistent might be originated from inherent defects in statistics. Therefore, we came back to the patient demographics and clinical parameters in Table 1, and combined with our professional knowledge, the results shown in Table 3 should be more reliable. That is to say, patients with traction holes were more likely to be suffered from RRD with CD when compared with patient with atrophic holes.

The incidence of CD in RRD patients was significantly higher in retinal breaks located posterior to the equator than in retinal breaks located anterior to or just on the equator, consistent with the analysis above. The retinal breaks located posteriorly, including macular hole, may be a potential risk factor for the development of CD in RRD patients. Patients with whole retinal detachment have a higher risk of developing CD than patients with partial retinal detachment, consistent with the broader exposure of the retinal pigment epithelium and more serious hypotony.

## Conclusions

his retrospective study of a large number of patients shows that hypotony, retinal breaks located posteriorly especially including macular hole, longer AL, and whole retinal detachment are potential risk factors for the development of CD in RRD patients. However, previously identified factors, old age and aphakic/pseudopakic eyes, were not confirmed. Future prospective studies should therefore be designed to provide additional evidence.

## Abbreviations

AEQ, anterior to equator, AL, axial length, BCVA, best-corrected visual acuity, CD, choriodal detachment, EQ, equator, IOP, intraocular pressure, PEQ, posterior to equator, PPV, pars plana vitrectomy, RRD, rhegmatogenous retinal detachment
